# Dual trigger improves response to ovarian stimulation and ICSI outcomes in patients with a previous r-hCG triggered ICSI cycle

**DOI:** 10.5935/1518-0557.20210065

**Published:** 2022

**Authors:** Amanda Souza Setti, Luis Guilherme Louzada Maldonado, Daniela Paes de Almeida Ferreira Braga, Assumpto Iaconelli Jr., Edson Borges Jr.

**Affiliations:** 1 Fertility Medical Group, São Paulo, SP, Brazil; 2 Instituto Sapientiae - Centro de Estudos e Pesquisa em Reprodução Humana Assistida, São Paulo, SP, Brazil

**Keywords:** ICSI, oocyte maturation, ovarian stimulation, implantation

## Abstract

**Objective:**

To evaluate if ovarian response to controlled ovarian stimulation (COS) and intracytoplasmic sperm injection (ICSI) outcomes are improved by the use of dual trigger (gonadotropin-releasing hormone (GnRH) agonists plus recombinant human chorionic gonadotropin (r-hCG)) in patients with previous cycles triggered with r-hCG.

**Methods:**

This case-control study included 88 matched cycles performed in 88 patients, which had the first ICSI cycle triggered with r-hCG (n=44), and the following ICSI cycle with dual trigger (n=44). We compared the cycle outcomes between the groups. In a second case-control within-subject analyses, we compared the ICSI outcomes between patients which had the first ICSI cycle triggered with r-hCG only (n=18), and the following ICSI cycle with dual trigger (n=18) or r-hCG only (n=18).

**Results:**

Upon investigating repeated cycles (r-hCG only vs. dual trigger), we found higher oocyte yield and mature oocyte rates, lower immature oocyte rates, higher fertilization rates, and higher blastocyst development rates; and higher rates of cycles with embryos transferred and implantation in the dual trigger cycle.

**Conclusions:**

The dual trigger regimen is a more effective approach than r-hCG trigger in patients with a previous r-hCG triggered ICSI cycle, yielding improved response to COS, and better laboratorial and clinical outcomes.

## INTRODUCTION

For assisted reproductive technology, we usually trigger final follicular maturation administering recombinant human Chorionic Gonadotropin (r-hCG) as a surrogate for the natural LH surge. More recently, gonadotropin-releasing hormone (GnRH) agonists have been applied to that end, primarily in high responder patients, to prevent early onset ovarian hyperstimulation syndrome (OHSS), due to the induction of more physiologic LH and FSH releases (Griesinger *et al*., 2006; Humaidan *et al*., 2011).

The combination of a GnRH agonist and hCG to trigger final oocyte maturation is called "dual trigger" (Shapiro *et al.*, 2008). The use of dual trigger to induce an endogenous LH surge has been explored in different scenarios. In patients with a history of immature oocytes, it seems to improve the incidence of mature oocytes (Griffin *et al*., 2014). The benefits of using a dual trigger have also been demonstrated in patients with repetitive immature oocytes retrieved, and empty follicle syndrome (Castillo *et al*., 2013). Zhang *et al*. (2017) reported a significantly higher number of retrieved oocytes, a higher percentage of mature oocytes, and better oocyte yield in dual trigger groups, compared with the conventional hCG trigger group. In addition, there were significantly improved pregnancy outcomes in normal responders (Lin *et al*., 2013), in women with diminished ovarian reserve (Lin *et al*., 2019), and in patients with a history of poor fertilization (Pereira *et al*., 2016; Elias *et al*., 2017). However, the advantage of using dual trigger in patients who previously failed an intracytoplasmic sperm injection (ICSI) cycle is still unclear.

The goal of this study was to compare ovarian response to controlled ovarian stimulation (COS) and ICSI outcomes among patients in whom final follicular maturation was achieved with the administration of dual trigger or standard r-hCG trigger.

## MATERIALS AND METHODS

### Experimental design, patients, inclusion and exclusion criteria

This case-control study included data obtained via chart review from 88 cycles matched by female age, performed in 88 patients undergoing ICSI between January and March 2019, in a private university-affiliated IVF center. The inclusion criteria were as follows: couples with primary infertility undergoing their first ICSI cycle, because of male factor infertility, whom had fresh embryo transfer on day 5 of embryo development.

We used rFSH for COS in all patients, and we achieved pituitary suppression with GnRH antagonist (cetrorelix acetate). We achieved final follicular maturation with the administration of GnRH agonist and r-hCG in the Dual Trigger Group (n=44), and r-hCG in r-hCG Only Trigger Group (n=44).

Ovarian response to COS and ICSI outcomes were compared between the groups. Primary endpoints were the number and maturity of retrieved oocytes.

**Figure 1:**
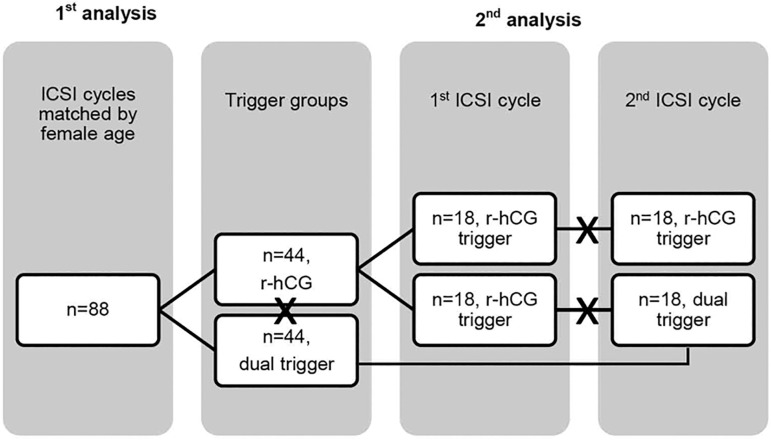
Study design Note: "X" marks the comparisons.

In a subsequent analysis, we ran a retrospective case-control within-subject analysis, to compare the response to COS and ICSI outcomes in patients who had the first ICSI cycle triggered with r-hCG only (r-hCG Group, n=18), and the following ICSI cycle triggered with GnRH agonist and r-hCG (Dual Trigger Group, n=18). We also compared the cycles' outcomes of patients that had two consecutive ICSI cycles triggered with r-hCG only (r-hCG 1^st^ Cycle Group, n=18 and r-hCG 2^nd^ Cycle Group, n=18) (Figure 1). For that, we matched the first ICSI cycles with those from the previous analysis, in order to verify if patients presenting with similar characteristics, who had their previous cycles triggered with r-hCG only would also benefit from a following cycle triggered with r-hCG only. The cycles were matched based on female age (± 1 y-old), mature follicular rate (± 5%), number of retrieved oocytes (± 2), mature oocyte rate (± 5%), blastocyst development rate (± 5%).

All patients signed a written informed consent form. The local Institutional Review Board approved the study.

### Controlled ovarian stimulation

On the third day of the cycle, we started controlled ovarian stimulation by the administration of r-FSH (Gonal-F, Serono, Geneva, Switzerland) daily doses. When at least one follicle ≥14 mm was seen, pituitary blockage was performed using gonadotropin-releasing hormone (GnRH) antagonist (GnRHa, Cetrotide^®^; Merck KGaA, Darmstadt, Germany). When three or more follicles attained a mean diameter of ≥17 mm and there were adequate serum estradiol levels, we triggered the final follicular maturation with the administration of a GnRH agonist (triptorelin 0.1 mg, Gonapeptyl; Ferring GmbH, Kiel, Alemanha) and r-hCG (250µg, Ovidrel^®^, Merck KGaA, Geneva, Switzerland) in the Dual Trigger Group or r-hCG (Ovidrel^®^) in the r-hCG-only Trigger Group. We retrieved the oocytes 35 hours later.

### Intracytoplasmic sperm injection, embryo culture and transfer

We performed intracytoplasmic sperm injection according to Palermo *et al*. (1992). We cultured the embryos in 50-µL drops of culture medium (Global®, LifeGlobal, Guilford, USA), covered with paraffin oil, in a humidified atmosphere, under 6% CO_2_, at 37ºC, for five days.

The blastocyst development rate was defined as the number of embryos that reached blastocyst stage at day five by the number of 2PN embryos in each cycle. On day 5, we transferred one to two embryos per patient, depending on maternal age and embryo quality, using a soft catheter under transabdominal ultrasound guidance.

### Clinical follow-up

Clinical pregnancy was confirmed upon detection of at least one intrauterine gestational sac with fetal heartbeat at 6-8 weeks of gestation. We calculated the pregnancy rates per transfer. Miscarriage was defined as pregnancy loss before 20 weeks of gestation.

### Data analysis and statistics

We compared the demographics between the groups using the Student's t-test. Follicles with a mean diameter of ≥17mm on the trigger day were considered mature. The number of mature follicles per total number of follicles on the trigger day.

We compared the ICSI outcomes between the groups using general linear models (GLM) followed by Bonferroni post hoc, adjusted for paternal age, as this variable was significantly different between the groups. The oocyte and embryo utilization rates were calculated as the sum of transferred and frozen embryos per retrieved oocytes or fertilized oocytes, respectively, and were compared between the groups using GLM, followed by Bonferroni post hoc, adjusted for paternal age. The main outcome measure was the ovarian response to COS.

Data are expressed as mean±standard deviation and *p*-values. *P*-value was significant at 5% (<0.05). We ran the analysis using SPSS Statistics 21 (IBM, New York, New York, USA).

## RESULTS

Table 1 describes the patients' demographics, response to COS and laboratorial ICSI outcomes. The Dual Trigger Group had a significantly higher number of retrieved oocytes, oocyte yield, and lower incidence of metaphase I compared to the r-hCG Trigger Group. There were no significant differences for other variables.

**Table 1.** Descriptive analysis of patients’ demographics, response to COS and laboratorial ICSI outcomes according to trigger groups matched by female age.

Variables	Dual trigger (n=44)	r-hCG trigger (n=44)	*p*-value
Female age (years)	37.27±3.94	37.91±3.80	0.443
Male age (years)	39.61±4.54	37.18±5.08	0.028
Female BMI	24.83±3.11	24.51±3.90	0.682
FSH dose (IU)	2685.26±829.53	2519.58±877.67	0.397
Length of stimulation (days)	10.24±1.37	10.92±1.71	0.082
Length of pituitary suppression (days)	4.49±1.03	4.64±1.19	0.584
Total number of follicles on trigger day	10.64±6.53	8.43±6.59	0.119
Mature follicular rate (%)	36.12±17.38	43.73±26.10	0.210
Estradiol level (pg/mL)	1685.77±992.52	1188.57±1009.53	0.155
Number of retrieved oocytes	7.89±5.31	5.30±5.07	0.022
Oocyte yield (%)	72.51±24.77	55.10±30.50	0.005
MII oocyte rate (%)	70.33±26.87	63.17±30.29	0.266
MI oocyte rate (%)	6.50±2.14	14.53±20.92	0.044
PI oocyte rate (%)	17.45±22.90	15.70±24.16	0.741
Fertilization rate (%)	84.27±29.63	77.58±35.03	0.373
Blastocyst development rate (%)	29.98±39.72	30.66±30.69	0.948

Note: values are mean ± standard deviation, unless otherwise noted. COS – controlled ovarian stimulation, r-hCG – recombinant human chorionic gonadotropin, BMI – body mass index, IU – international unit, MII – metaphase II, MI – metaphase I, PI – prophase I.

The oocyte utilization rate (26.11%±4.10 *vs*. 27.37%±4.10, *p*=0.829) and the embryo utilization rate (48.45±5.34 *vs*. 60.75±6.09, *p*=0.136) were similar between the Dual Trigger Group and the r-hCG Trigger Group, respectively.

There was a higher number of cycles with embryo transferred in the Dual Trigger Group, compared to the r-hCG Trigger Group 78.57% (33/44) *vs*. 57.14% (24/44), *p*=0.035. The groups had similar rates of implantation (23.74±39.54 *vs*. 27.27±45.58, *p*=0.761), clinical pregnancy (10/33, 30.30% *vs*. 8/24, 33.33, *p*=0.808), and miscarriage (1/10, 10.0% *vs*. 1/8, 12.5%, *p*=0.999), respectively.

In the subsequent case-control within-subject analysis; upon investigating repeated cycles (r-hCG only *vs*. dual trigger), there were higher oocyte yield, higher mature oocyte rate, lower immature oocyte rate, higher fertilization rate, and higher blastocyst development rate in the Dual Trigger Group compared to the r-hCG Trigger Group (Table 2).

**Table 2.** Descriptive analysis of patients’ demographics, response to COS and laboratorial ICSI outcomes in repeated cycles.

Variables	r-hCG Trigger (n=18)	Dual Trigger (n=18)	*p*-value
Female age (years)	37.83±0.77	38.22±0.77	0.721
Male age (years)	39.06±0.93	39.44±0.93	0.767
Female BMI	24.66±0.58	25.33±0.58	0.414
FSH dose (IU)	2875.00±190.44	2625.00±201.99	0.368
Length of stimulation (days)	10.41±1.33	10.47±1.70	0.911
Length of pituitary suppression (days)	4.76±0.83	4.53±1.23	0.518
Total number of follicles on trigger day	11.17±1.799	10.56±1.80	0.810
Mature follicular rate (%)	46.63±20.30	34.54±21.23	0.095
Estradiol level (pg/mL)	1246.67±261.21	1431.22±301.62	0.644
Number of retrieved oocytes	7.17±1.36	7.72±1.36	0.772
Oocyte yield (%)	60.83±3.18	84.38±3.25	<0.001
MII oocyte rate (%)	59.50±2.44	74.29±3.26	<0.001
MI oocyte rate (%)	18.22±2.63	5.80±2.55	<0.001
PI oocyte rate (%)	16.11±1.34	10.00 ± 0.95	<0.001
Fertilization rate (%)	80.00±2.83	88.12±2.35	0.027
Blastocyst development rate (%)	18.21±1.14	41.67±2.63	<0.001

Note: Retrospective case-control within-subject analysis performed to compare the response to COS and ICSI outcomes in patients who had the first ICSI cycle triggered with r-hCG only and the following ICSI cycle triggered with GnRH agonist and r-hCG. Values are mean ± standard error, unless otherwise noted. COS – controlled ovarian stimulation, ICSI – intracytoplasmic sperm injection, r-hCG – recombinant human chorionic gonadotropin.

The oocyte utilization rate tended to be higher in the Dual Trigger Group compared to the hCG Trigger Group (32.31%±3.46 *vs*. 17.68%±1.09, *p*=0.074, respectively). The embryo utilization rate was significantly higher in the Dual Trigger Group compared to the hCG Trigger Group (59.04%±2.06 *vs*. 38.59%±1.87, *p*<0.001, respectively).

A higher number of cycles with embryo transfer (16/18, 88.89% *vs*. 10/18, 55.55%, *p*=0.026) and a higher implantation rate (26.92%±1.44 *vs*. 10.00%±1.00, *p*<0.001) were seen in the Dual Trigger 2^nd^ Cycle Group compared to the r-hCG-only trigger 1^st^ Cycle Group, respectively. The groups had similar rates of clinical pregnancy (7/16, 43.75% *vs*. 1/10, 10.00%, *p*=0.099), and miscarriage (1/7, 14.29% *vs*. 1/1, 100%, *p*=0.250), respectively.

Upon investigating repeated cycles (r-hCG only vs. r-hCG only), there were lower immature oocyte rate and higher implantation rate in the r-hCG Trigger 2^nd^ Cycle Group compared to r-hCG Trigger 1^st^ Cycle Group (Table 3).

**Table 3.** Descriptive analysis of patients’ demographics, response to COS and laboratorial ICSI outcomes in repeated cycles.

Variables	r-hCG Trigger 1^st ^Cycle (n=18)	r-hCG Trigger 2^nd^ Cycle (n=18)	*p*-value
Female age (years)	37.44±0.14	37.44±0.14	>0.999
Male age (years)	38.72±1.65	39.11±1.65	0.868
Female BMI	24.72±0.95	25.00±0.95	0.833
FSH dose (IU)	2620.59±154.62	2841.67±150.26	0.305
Length of stimulation (days)	10.41±1.70	10.94±1.75	0.377
Length of pituitary suppression (days)	4.88±1.17	4.88±1.17	>0.999
Number of follicles	10.00±2.44	12.50±2.44	0.468
Mature follicular rate (%)	44.97±26.90	35.57±18.26	0.242
Estradiol level (pg/mL)	1520.31±227.97	1556.69±322.40	0.927
Number of retrieved oocytes	6.00±1.87	9.56±1.87	0.178
Oocyte yield (%)	61.81±2.62	72.23±2.62	0.190
MII oocyte rate (%)	62.41±2.63	65.54±2.63	0.686
MI oocyte rate (%)	17.00±1.30	12.72±1.08	0.011
PI oocyte rate (%)	9.09±0.91	10.00±0.79	0.450
Fertilization rate (%)	66.25±2.88	67.92±2.38	0.655
Blastocyst development rate (%)	19.75±2.14	34.51±1.79	0.145

Note: Retrospective case-control within-subject analysis performed to compare the response to COS and ICSI outcomes in patients who had two consecutive ICSI cycles triggered with r-hCG only. Values are mean ± standard error, unless otherwise noted. COS – controlled ovarian stimulation, ICSI – intracytoplasmic sperm injection, r-hCG – recombinant human chorionic gonadotropin.

The oocyte utilization rate (30.21%±1.55 *vs*. 31.75%±1.70, *p*=0.975) and the embryo utilization rate (64.03±2.56 *vs*. 46.02%±2.02, *p*=0.098) were similar between the r-hCG Trigger 1^st^ Cycle Group and the r-hCG Trigger 2^nd^ Cycle Group, respectively.

Similar number of cycles with embryo transferred (11/18, 61.11% *vs*. 13/18, 72.22%, *p*=0.725) and clinical pregnancy rate (3/11, 27.27% *vs*. 0/13, 0.00%, *p*=0.099) were seen between the groups. There were no miscarriages in both groups.

## DISCUSSION

In this study, we found that patients with a previous r-hCG triggered ICSI cycle benefited from a dual trigger in the following ICSI cycle. Our results are in agreement with several previous studies reporting higher number of collected oocytes (Lin *et al*., 2013) and mature oocytes (Lin *et al*., 2013), higher top-quality embryo rate (Decleer *et al.*, 2014), increased implantation (Lin *et al*., 2013) and pregnancy rates (Lin *et al*., 2013) in patients with a normal response to COS, when dual trigger was performed compared to conventional hCG trigger.

Previous studies performed in poor responder patients also demonstrated that dual trigger yields higher numbers of retrieved oocytes (Oliveira *et al*., 2016), more mature oocytes retrieved (Griffin *et al*., 2014; Zilberberg *et al*., 2015; Oliveira *et al*., 2016), higher fertilization (Oliveira *et al*., 2016) and top-quality embryo rates (Zilberberg *et al*., 2015), higher implantation (Oliveira *et al*., 2016) and pregnancy rates (Schachter *et al*., 2008), and higher live-birth rate (Oliveira *et al*., 2016).

Differences in the results of the aforementioned studies might be attributed to the GnRH analogue used for pituitary suppression, origin and dose of the hCG administered. In fact, it has been shown that GnRH agonist for trigger after the use of GnRH antagonist for pituitary suppression increases implantation rates (Schachter *et al*., 2008). This happens because, initially, the GnRH antagonist blocks endometrial GnRH receptors. After the administration of GnRH antagonist as trigger of final oocyte maturation, the GnRH antagonist is displaced from the endometrial receptors, improving endometrial receptivity (Schachter *et al*., 2008).

The use of dual trigger with GnRH agonist and hCG to induce final oocyte maturation combines the advantages of both hormone regimens with (i) the induction of a physiological release of FSH through the FSH peak induced by the GnRH agonist, and (ii) improving luteal phase recruitment (Shapiro *et al*., 2011). These issues will be discussed below.

The induction of an FSH peak by the GnRH agonist in the dual trigger, in addition to the LH surge, has been shown to increase the number of mature oocytes (Zeleznik *et al*., 1974; Richards *et al*., 1976; Lamb *et al*., 2011; Castillo *et al*., 2013; Griffin *et al*., 2014). FSH surge is associated with oocyte maturation, expansion of cumulus cells and release of proteolytic enzymes involved in the ovulation process (Yding Andersen, 2002; Richards *et al*., 2005).

The GnRH-a trigger down-regulates the pituitary gland and reduces LH to a level that is insufficient for maintaining corpus luteum function (Shapiro & Andersen, 2015). The GnRH triggering of final oocyte maturation is associated with unfavorable effects on endometrial receptivity that can happen due to a direct effect, dysfunctional corpus luteum, or early luteolysis. Several studies have showed smaller midluteal phase ovarian volume (Engmann *et al*., 2008; Garcia-Velasco *et al*., 2010), significantly lower luteal phase steroidal concentration and peptides (Engmann *et al*., 2008), and shorter duration of the luteal phase in non-supplemented cycles (Garcia-Velasco *et al*., 2010) using the GnRH agonist trigger. This evidence supports corpus luteum dysfunction after GnRHa trigger. One of the consequences of dysfunctional corpus luteum is a potential injurious impact on endometrial receptivity. In fact, significantly higher miscarriage rates and lower ongoing pregnancy rates after GnRHa trigger have been reported (Humaidan *et al*., 2005; Kolibianakis *et al*., 2005). On the other hand, hCG-trigger causes the production of endogenous progesterone that stimulates uterine changes for successful implantation.

The results here presented might provide another tool for the clinician to use in the trigger-regimen decision-making process. However, this is a retrospective study with its inherent limitations and bias. This study was limited by its small sample size.

In conclusion, the dual trigger regimen is a more effective approach than the r-hCG trigger in patients with a previous r-hCG triggered ICSI cycle, yielding improved ovarian response and ICSI outcomes.
